# Does Accountability Require Agency? Comment on Responsibility and Accountability in the Algorithmic Society

**DOI:** 10.1007/s13347-025-01014-z

**Published:** 2026-01-12

**Authors:** Tillmann Vierkant

**Affiliations:** School of Philosophy, Psychology and Language Sciences, University of Edinburgh, Edinburgh, UK

**Keywords:** Accountability, Responsibility, Proleptic accounts, Buck stopping, Agency

## Abstract

In their intriguing paper *Responsibility and Accountability in an Algorithmic Society (2025)* the authors argue that the debate on how to deal with responsibility related issues with algorithmic agents requires a distinction between responsibility and accountability. In this comment to their paper, it is argued that while the notion of accountability as understood by the authors brings some significant benefits it also is ambiguous in an important way. Accountability could be understood as being purely instrumental with regard to general morally desirable consequences or it could be understood as necessarily containing an element of scaffolding for the agent who is held to account. The comment develops the options and discusses the consequences of choosing either of them.

In their intriguing paper *Responsibility and Accountability in an Algorithmic Society (2025)* the authors argue that the debate on how to deal with responsibility related issues with algorithmic agents requires a distinction between responsibility and accountability. The key idea behind the distinction is that accountability provides rule-based frameworks which govern collectives and institutions,^1^ while responsibility practices concern interpersonal behaviour, involve blame, and are driven by reactive attitudes.

The authors argue that their distinction can make progress with three problems for AI accountability identified by Nissenbaum (1996). The first, the problem of many hands, is usually understood to be the fact that it is hard to hold anyone specific to account when AIs create harms because the human agency behind the AI is too distributed between programmers, designers, users etc. The second problem is that AIs are what they call ‘buggy’. They are very complex systems and when things go wrong it is easy to argue that it was not the fault of the human, but that there might have been a bug in the code. The third problem is computer scapegoating. AIs are so autonomous that it seems unfair to hold humans responsible for their autonomous decisions. Unfortunately, it is also hard to see that AIs themselves really could be full appropriate targets for blame. Together, these problems lead to so-called responsibility gaps (Matthias, 2004), i.e. cases where intuitively someone should be responsible, but because of these issues, nobody seems to be.

By divorcing accountability from responsibility, the authors argue that they can make progress with all three problems. On their account, it is possible to hold humans in the causal chain accountable, even though they are not responsible for the harm. The account is designed to deal with the problems generated by AI, but it is also useful in the much wider and often equally problematic field of collective accountability.

Their account of accountability allows that certain role holders in institutions can have ‘buck stopping’ functions, even if there are no grounds to blame an individual. The CEO in a company might have to answer questions, issue apologies and leave her post, even if she is not personally responsible for the harmful behaviour of an AI or individual in the company she leads.

Conversely, the authors argue that their account is also beneficial for responsibility practices because it makes it easier to accept that problems like many hands are responsibility-attenuating, while keeping accountability in place. Furthermore, the authors argue that their distinction makes for better responsibility practices in companies, because these are important in circumstances where codified accountability might be too costly, or where there are no accountability structures because the problem is new, small or niche.

This solution is ingenious, but there also important limitations to the proposal, which are perhaps not immediately obvious. The first worry is a terminological one, but quite a number of substantive issues are connected with the terminology. The authors have chosen to call their rules-based framework ‘accountability’, but this is slightly confusing because in the literature, accountability is often understood as what the authors call ‘consequences’, i.e. one facet of responsibility that deals with the fittingness of sanctions like eg expression of reactive attitudes or public shaming (consequences) for blameworthy actions (Shoemaker, 2011, 2020). The authors are aware of this and in a footnote say that they do not want to use the term ‘accountability’ to refer to consequences, because it would lead to confusion with their preferred notion of accountability. While this is obviously a fair point, it is nevertheless a slight worry that accountability in their sense is similar but importantly different to the notion of accountability in most of the literature. This has the potential to be misleading and the paper is in danger of being an instance of this.

As already mentioned, the authors argue that a key payoff of their distinction is that it allows to make progress with the three barriers to accountability pointed out by Nissenbaum (1996). But this is slightly misleading, because Nissenbaum understands accountability in the traditional sense (i.e. what the authors call consequences). As the authors understand accountability differently, it is not obvious that their framework does address the worries from the three barriers in the Nissenbaum sense, and it is also not clear how worried we need to be about these barriers for accountability as they understand it in the first place.

To a degree, perhaps, this does not need to be a big problem. The authors have clearly provided a helpful tool that allows at least for some form of accountability in cases where establishing responsibility is difficult or impossible and it might be demanding too much to insist that their framework solve all the intractable issues associated with responsibility gaps.

However, the ambiguity that the term ‘accountability’ carries because of its history is also felt in more substantive issues the authors discuss.

Most important in this context is a certain ambiguity in the justification of accountability practices with regard to how important an agentive contribution of the person to be held accountable for some harm to that harm is.

On the one hand, the authors seem to say that the agentive contribution does not matter very much. In fact, this is exactly what gives their framework its bite when it comes to buck-stopping. Accountability is due as long as there is a rule that in case of some harm a role holder specified by the rule has to face the appropriate sanctions. This is true even if the role holder has done everything one could reasonably expect of them to prevent this harm and is therefore not to blame for the outcome.

On the other hand, the authors insist that:

*[…] holding an unrelated citizen on a different continent accountable for the behavior of a system would make little sense. An agent who is held accountable must have a connection to the action they are held accountable for, even if that connection is more tenuous than would be required for full-fledged responsibility. (*Fleisher et al., 2025*, p.26).*

Their reasoning for this limitation is that they justify accountability on the grounds of its positive moral effects. Having clear accountability rules in a company e.g. means that role holders will be extra careful, which will have positive overall effects and make harm less likely in the longer term. At the same time, it seems reasonable to insist that holding someone unrelated on a different continent accountable is not going to generate this positive effect.

However, this limitation does not rule out other, more plausible, cases where the punished has no agency with regard to the harm. The authors themselves discuss collective punishment. An organisation could have the rule that when some harm occurs, everyone in the whole collective, whether they have any agency regarding that harm or not, will be punished. The authors admit that there could be instances where such punishment will have positive effects but insist that the practice as a whole would be unfair and would lower morale and because of that, would not bring the desired positive long-term effects.

We can start examining this argument by focussing on an interesting and perhaps surprising term the authors use. The authors claim that such a practice would be perceived as ‘unfair’. Yet they do not elaborate on why that would be, but it is a bit surprising that they assume that the notion of fairness is unproblematic here. After all, fairness is normally understood as being treated according to what one deserves and their account of accountability is supposed to be independent of richer notions of desert. Now obviously, they could insist that all that fairness means is that one is treated according to the rules, but in that case, the collective punishment is fair and an agent who willingly accepted a role that comes with that rule cannot complain that they have been treated unfairly in this sense.

In fact, there are many examples in real life where we think such collective punishments are absolutely unproblematic. Think for example of team fouls in basketball. In this case, a team is punished as a whole for the fouls of individuals, and it seems right that punishment is also meted out to players who have not committed any fouls, because they are members of the offending team. If a company is held accountable for a harm and has to pay compensation, it means that all employees – including the cleaning staff who had no agency that could have helped to avoid the harm – will not get a pay rise.

The hope with such punishments is likely that they will have an overall positive effect down the line. They are justified in quite similar instrumentalist ways to the accountability practices the authors describe. They will make everybody who has agency more careful, both because of their own risk, but also out of solidarity with the cleaning staff and with rule-abiding basketball team members.

As the authors rightly point out, we also don’t need to be too worried about people being punished too severely despite the fact that they do not deserve it in any richer sense that amounts to more than ‘thems the rules’ (Vargas, 2013). This is because accountability in the authors’ sense of the word has a built-in limit to what punishments can be appropriate, given the fact that the agents might incur these punishments without any blame. Dismissal seems like it can be appropriate, jail does not.

However, it is perfectly understandable that the authors are reluctant to embrace accountability without any agency that could avoid the harm. In the world of responsibility, there is a space between being deserving of blame in a strong sense and having made no agentive contribution to an outcome. The existence of this space explains the intuition that we can be justified in feeling that fairness in a richer sense is an issue, even in cases where no one is to blame. We might well feel that it is fairer to hold someone with agency to account than someone without, but this is because we tacitly assume that for such an agent our responsibility practices are more adequate than for someone who had no agency in the matter, because they can benefit from these practices in ways which someone who had no agency cannot.

As Victoria McGeer (2019, see also McGeer & Pettit, 2015) has pointed out, the debate on justifications of responsibility is often incredibly time-slice fixated: i.e. did the agent have the right control to avoid the harm *when it happened?* If we accept that responsibility practices are about scaffolding agents rather than just deserts, then it is easy to see why we need to make the difference between the agent that has been agentially involved in the harm and some unconnected person.^2^ It is likely that something in the agency of the involved agent has somehow gone wrong, even if we could not reasonably expect them to avoid the harm and our responsibility practices will help the agent to avoid similar harms in the future.

But obviously, the authors are not proposing an account of proleptic responsibility, but an account of the distinct phenomenon of accountability. So, should they import that dimension of proleptic responsibility into their account of accountability?

The advantage of steering clear of borrowing from proleptic responsibility for their account is that it makes their notion of accountability cleaner. According to their framework, accountability is simply about the general positive consequences of explicit accountability rules. Justifications that focus on scaffolding agents to become better agents doesn’t cleanly fit into that framework and also undermine the distinction between accountability and proleptic responsibility.

But the disadvantage is that it also means that their account is a lot less intuitive (because it is hard to see why bystanders could not be accountable) and can only make a quite moderate contribution in reducing responsibility gaps (because it is harder for the harm causing agents to accept that they are appropriate targets).

Both options seem perfectly legitimate and as discussed bring advantages and disadvantages, but it is not clear to me that the authors can hold on to the claim that agency matters and collective punishments are never justified if they opt for the cleaner option that accountability really is just about the positive consequences that sanctioning rule breaking brings.

## Data Availability

Not applicable.

## Footnotes

1 Accountability rules are explicit rules of collectives but they are not the same as legal rules. This is why accountability is distinct from liability according to the authors.

2 There have been suggestions that there is room between a bystander and agent for what Tollon & Vallor (2025) call entangled agents i.e. agents who have no direct agency but are entangled with the agent who does create the harm. Examples might be an agent who feels responsible for the war crimes of their country or the behaviour of their dog. There is no space to discuss this fascinating approach in detail here but to me it seems clear that these entanglement cases fall in two quite different camps. On the one hand there is the dog case where the owner has a lot of agency over the behaviour of the dog just not at the moment when the dog misbehaves. These seem like cases where agency scaffolding is highly appropriate. In other cases where the agent really has no or minimal agency with regard to the harm it seems to me that the talk of responsibility here is much looser and describes a different phenomenon.
